# Cytometric analysis of patients with COVID-19: what is changed in the second wave?

**DOI:** 10.1186/s12967-021-03072-1

**Published:** 2021-09-23

**Authors:** Giulia Scalia, Maddalena Raia, Monica Gelzo, Sara Cacciapuoti, Annunziata De Rosa, Biagio Pinchera, Riccardo Scotto, Agnese Giaccone, Mauro Mormile, Gabriella Fabbrocini, Ivan Gentile, Roberto Parrella, Giuseppe Castaldo

**Affiliations:** 1 CEINGE-Biotecnologie Avanzate, via Gaetano Salvatore 486, 80145 Naples, Italy; 2 Dipartimento di Medicina Molecolare e Biotecnologie Mediche, Università di Napoli Federico II, Naples, Italy; 3 Dipartimento di Medicina Clinica e Chirurgia, Università di Napoli Federico II, Naples, Italy; 4 Dipartimento di Malattie Infettive e Emergenze Infettive, Divisione di malattie infettive respiratorie, Ospedale Cotugno, AORN dei Colli, Naples, Italy

**Keywords:** Cytometry, Interleukin-6, Lymphocytes, Targeted therapy, COVID-19

## Abstract

**Background:**

Coronavirus disease 2019 (COVID-19) pandemic had a 1st wave in Europe from March to May 2020 and a 2nd wave since September 2020. We previously studied 35 hospitalized COVID-19 patients of the 1st wave demonstrating a cytokine storm and the exhaustion of most lymphocyte subpopulations. Herein, we describe the results obtained from COVID-19 patients of the 2nd wave.

**Methods:**

We analyzed interleukin (IL)-6 by human-specific enzyme-linked immunosorbent assay and a large set of lymphocyte subpopulations by flow cytometry in 274 COVID-19 patients hospitalized from September 2020 to May 2021.

**Results:**

Patients of 2nd wave compared with those of 1st wave showed lower serum IL-6 levels and a higher number of B and most T lymphocyte subpopulations in advanced stages, in relation with the age and the gender. On the other hand, we observed in 2nd wave patients: (i) a reduction of most lymphocyte subpopulations at mild and moderate stages; (ii) a reduction of natural killer cells and T regulatory cells together with a higher number of activated T helper (TH) 17 lymphocytes in all stages, which were mainly related to steroid and azithromycin therapies before hospitalization.

**Conclusions:**

COVID-19 had a less severe impact in patients of the 2nd wave in advanced stages, while the impact appeared more severe in patients of mild and moderate stages, as compared with 1st wave patients. This finding suggests that in COVID-19 patients with milder expression at diagnosis, steroid and azithromycin therapies appear to worsen the immune response against the virus. Furthermore, the cytometric profile may help to drive targeted therapies by monoclonal antibodies to modulate specific IL/lymphocyte inhibition or activation in COVID-19 patients.

## Introduction

Coronavirus disease 2019 (COVID-19) may appear with a widely variable clinical expression, from asymptomatic or mild [1] to severe forms [2] with acute respiratory distress syndrome (ARDS) and multi-organ failure in less than 10% of patients. The COVID-19 pandemic had a 1st wave in Italy from March to May 2020. To cast light on immunological and cytological response, we performed a preliminary study on 35 patients hospitalized for COVID-19 demonstrating a reduction of lymphocytes including both B and T populations. Among T lymphocytes, we observed a reduction of helper, suppressor and regulatory subpopulations, in relation with the disease severity [3]. Serum IL-6 was increased in most patients with a significant association with the disease severity. Thus, the analysis demonstrated the picture of lymphocyte exhaustion induced by the cytokine storm. The molecular mechanisms that cause these complications only in a small subset of COVID-19 patients are still under study [4]. In fact, even if most lymphocyte subpopulations are depleted, some T cell subgroups, involved in the synthesis of cytokines, are strongly activated in few severe COVID-19 patients [5, 6], creating a vicious circle between the cytokine storm and lymphocyte exhaustion [7]. Thus, the analysis of lymphocyte subpopulations in COVID-19 patients could contribute to predict the outcome and to define personalized therapies targeting different cell or cytokine pathways on the basis of cytometric analysis [3, 8, 9].

After a lockdown during summer 2020, the pandemic in Italy had a 2nd wave since September 2020. The two pandemic waves had several differences [10–12]. In fact, during the 1st wave most patients were diagnosed after the onset of symptoms by molecular analysis on nasopharyngeal swab that often required 2 or 3 days, and the patients were hospitalized soon, after the result. While, most patients of the 2nd wave were diagnosed when they were still asymptomatic because they had been traced following a contact with a COVID-19 patient. The result of the nasopharyngeal test was obtained more rapidly and some patients began to be treated with different combinations of steroids, azithromycin, and heparin several days before hospitalization [13–15]. During the 2nd wave, we studied 274 novel COVID-19 patients recovered in our specialized hospitals with the same protocol of the first study [3]. We now describe the results, in comparison with those obtained in patients from the 1st wave.

## Methods

### Patients

All 274 consecutive adult patients (mean age: 61.2 years, range: 17–91 years; 135 females, 49.0%) with a diagnosis of COVID-19 admitted at one of our hospitals from September 2020 to May 2021 (2nd wave of COVID-19 pandemic) were enrolled. The lone exclusion criterion was the refusal or the impossibility to sign the informed consent. None of the patients admitted to our Institutions during the period of our study was excluded. The diagnosis of COVID infection was confirmed by molecular analysis on nasopharingeal swab [16]. All the enrolled patients were classified on the basis of the seven ordinal scale made by the World Health Organization (WHO)-Research and Development Blueprint expert group and used in previous influenza studies [17, 18]. Furthermore, for each patient we recorded the assumption of drugs (particularly steroids, azithromycin or both drugs) before hospitalization. As shown in Table 1, 161/274 (59.0%) patients were treated with one or both the drugs before hospitalization.

**Table 1:** Steroids and/or azithromycin therapies before hospitalization in 2nd wave patients

Subgroups	Not treated	Only steroids	Only azithromycin	Steroids and azithromycin
WHO 3	53 (81)	5 (8)	2 (3)	5 (8)
WHO 4	44 (31)	21 (15)	6 (4)	70 (50)
WHO 5–7	16 (24)	13 (19)	5 (7)	34 (50)

N (%)

### Lymphocyte subpopulations and serum IL-6 analyses

Whole blood samples were collected at admission in tubes containing EDTA and then immediately analyzed by flow cytometry. Serum samples were separated from blood cells after the collection in tubes without anticoagulant and stored at − 80 °C until IL-6 analysis [3]. Immunophenotyping analysis was performed by multicolour flow cytometry [3]. Serum IL-6 levels were analyzed using human-specific enzyme-linked immunosorbent assay (ELISA) Max™ Set Deluxe kits (BioLegend, Inc., San Diego, USA), in accordance with the manufacturer's instructions.

### Statistical analysis

The data obtained in the 274 patients of the 2nd wave were compared to those collected in patients of the 1st wave [3]. Continuous data were reported as median and interquartile range (IQR). Comparisons between two groups were evaluated by Mann–Whitney U test. Statistical differences between three groups were assessed by Kruskal–Wallis test and Mann–Whitney U test as post-hoc test. Categorical data were reported as frequency and percentage. The chi-square test was used to compare the frequency of categorical variables between the groups. Linear regression analysis was used to assess the effects of age, gender and therapies on lymphocyte subpopulations. Statistical analysis was performed by SPSS (version 26, IBM SPSS Statistics). Graphics have been performed by KaleidaGraph software (version 4.5.4, Synergy, Reading, PA, USA). P values < 0.05 were considered as significant.

## Results

As shown in Table 2, the WHO stage distribution of COVID-19 patients of the two waves was not significantly different. In contrast, the age of patients of the 2nd wave was significantly lower, even if in both waves the age of patients increased (significantly in patients of the 2nd wave) with parallel to the WHO stage. Furthermore, although among patients of the 2nd wave we observed a significantly lower number of males as compared to the 1st wave, the percentage of males increased with the progression of the WHO stage.

**Table 2:** Comparison of demographics, serum IL-6 (pg/mL) and circulating lymphocytes (N/mmc) in COVID-19 patients of 1st wave and patients of 2nd wave at admission with different severity according to worst WHO stage for each patient

	Wave	All	WHO 3	WHO 4	WHO 5–7	Kruskal- Wallis
N (%)	1st	35	7 (20)	20 (57)	8 (23)	–
	2nd	274	65 (24)	141 (51)	68 (25)	–
	1st *vs* 2nd	–	n.s	n.s	n.s	
Age, yrs	1st	62 (50–73)	60 (39–62)	64 (51–73)	75 (57–79)	n.s
	2nd	55 (37–66)	34 (29–48)	59 (41–68) ^**a**^	57 (48–73)^b^	** < 0.0001**
	1st *vs* 2nd	**0.023**	n.s	n.s	n.s	
Males, N (%)	1st	27 (77)	4 (57)	16 (80)	7 (88)	–
	2nd	139 (51)	15 (23)	77 (55)	47 (69)^b^	–
	1st *vs* 2nd	**0.003**	n.s	**0.031**	n.s	
IL-6	1st	171 (90–397)	130 (90–223)	197 (86–375)	292 (53–769)^b^	**0.021**
0–4.5	2nd	28 (23–41)	28 (23–41)	28 (21–41)	31 (25–46)	n.s
	1st *vs* 2nd	** < 0.0001**	**0.0007**	** < 0.0001**	**0.004**	
Total	1st	1116 (539–1387)	1595 (588–1924)	1153 (653–1333)	450 (267–1033)	n.s
1500–3000	2nd	1058 (709–1683)	1289 (874–1860)	1058 (736–1577)	770 (518–1735)	n.s
	1st *vs* 2nd	n.s	n.s	n.s	n.s	
T	1st	793 (395–1027)	959 (306–1673)	801 (485–1015)^a^	309 (221–776)^b^	**0.041**
605–2460	2nd	801 (465–1245)	996 (600–1477)	780 (495–1187)	564 (336–1203)	n.s
	1st *vs* 2nd	n.s	n.s	n.s	n.s	
T helper	1st	482 (225–692)	692 (223–803)	527 (268–616)	215 (162–421)^b^	**0.050**
493–1666	2nd	461 (283–797)	539 (321–873)	461 (316–794)	364 (173–702)^b, c^	**0.040**
	1st *vs* 2nd	n.s	n.s	n.s	n.s	
T suppressor	1st	201 (109–357)	304 (56–482)	197 (123–357)	88 (30–310)	n.s
229–1112	2nd	249 (146–395)	287 (204–436)	236 (148–361)	198 (102–376)^b^	**0.018**
	1st *vs* 2nd	n.s	n.s	n.s	n.s	
B	1st	76 (40–154)	154 (47–241)	67 (37–119)	68 (38–106)	n.s
﻿72–520	2nd	141 (67–244)	106 (64–225)	160 (77–247)	121 (47–272)	n.s
	1st *vs* 2nd	**0.004**	n.s	**0.009**	n.s	
Naïve	1st	463 (287–915)	861 (412–1231)	468 (337–811)	252 (191–788)	n.s
126–1121	2nd	648 (431–1071)	759 (510–1180)	688 (440–1008)	482 (328–1077)	n.s
	1st *vs* 2nd	n.s	n.s	n.s	n.s	
NK	1st	134 (84–239)	153 (117–239)	148 (99–266)	88 (71–128)	n.s
﻿73–654	2nd	81 (48–132)	93 (51–157)	77 (47–125)	80 (48–130)	n.s
	1st *vs* 2nd	**0.004**	n.s	**0.002**	n.s	
T regulatory	1st	17.6 (7.0–27.3)	24.5 (17.6–35.1)	18.9 (8.4–31.4)	9.1 (3.2–17.1)^b^	**0.03**
7-52	2nd	9.1 (4.8–15.5)	11.0 (7.3–18.3)	9.3 (4.7–15.4)^a^	7.1 (2.96–12.7)^b^	** < 0.0001**
	1st *vs* 2nd	**0.002**	**0.024**	**0.008**	n.s	
Total activated	1st	137 (88–234)	234 (100–478)	137 (113–209)	87 (53–223)	n.s
86-799	2nd	193 (104–311)	171 (99–309)	206 (127–311)	138 (85–324)	n.s
	1st *vs* 2nd	n.s	n.s	**0.034**	n.s	
Activated T	1st	30.1 (11.8–56.9)	37.1 (6.1–98.9)	31.3 (19.6–54.5)	10.4 (7.2–61.9)	n.s
﻿14–411	2nd	25.0 (12.6–45.8)	24.7 (12.0–46.6)	31.0 (15.6–48.6)	19.9 (11.0–35.1)	n.s
	1st *vs* 2nd	n.s	n.s	n.s	n.s	
TH1	1st	93 (49–195)	180 (56–249)	89 (51–192)	44 (19–107)^b^	**0.050**
﻿37–220	2nd	79 (42–147)	99 (65–178)	80 (44–139)	55 (27–117)^b, c^	**0.016**
	1st *vs* 2nd	n.s	n.s	n.s	n.s	
Activated TH1	1st	6.3 (2.7–12.2)	6.4 (2.4–15.7)	6.2 (2.7–12.4)	5.3 (1.3–8.9)	n.s
0–20﻿	2nd	3.6 (1.7–7.6)	4.0 (1.6–6.9)	4.4 (2.0–10.3)	3.1 (1.6–6.1)	n.s
	1st *vs* 2nd	n.s	n.s	n.s	n.s	
TH17	1st	44.2 (22.3–65.1)	55.4 (22.3–66.0)	50.4 (27.6–66.1)	22.8 (12.0–42.0)	n.s
3.76–60.0	2nd	45.2 (24.1–76.6)	46.3 (19.6–70.6)	50.3 (26.1–80.8)	38.0 (20.6–76.9)	n.s
	1st *vs* 2nd	n.s	n.s	n.s	n.s	
Activated TH17	1st	0.57 (0.23–0.80)	0.69 (0.45–0.80)	0.55 (0.15–0.89)	0.40 (0.24–0.73)	n.s
﻿0–1.3	2nd	0.63 (0.00–1.44)	0.53 (0.00–1.29)	0.71 (0.11–1.66)	0.58 (0.00–1.76)	n.s
	1st *vs* 2nd	n.s	n.s	n.s	n.s	
Activated TH1/TH17	1st	12 (5.8–38)	10 (6.0–29)	12 (5.2–98)	11 (3.9–31)	n.s
	2nd	7.0 (3.0–27)	8.0 (2.2–92)	7.3 (3.5–35)	6.5 (3.0–16)	n.s
	1st *vs* 2nd	n.s	n.s	n.s	n.s	

Median and interquartile range. For each parameter we report the reference range

*n.s.* not significant

Significant values are reported in bold

^a^p < 0.01, WHO 4 *versus* WHO 3; ^b^p < 0.01, WHO 5–7 *versus* WHO 3; ^c^p < 0.01, WHO 5–7 *versus* WHO 4

**Fig. 1:**
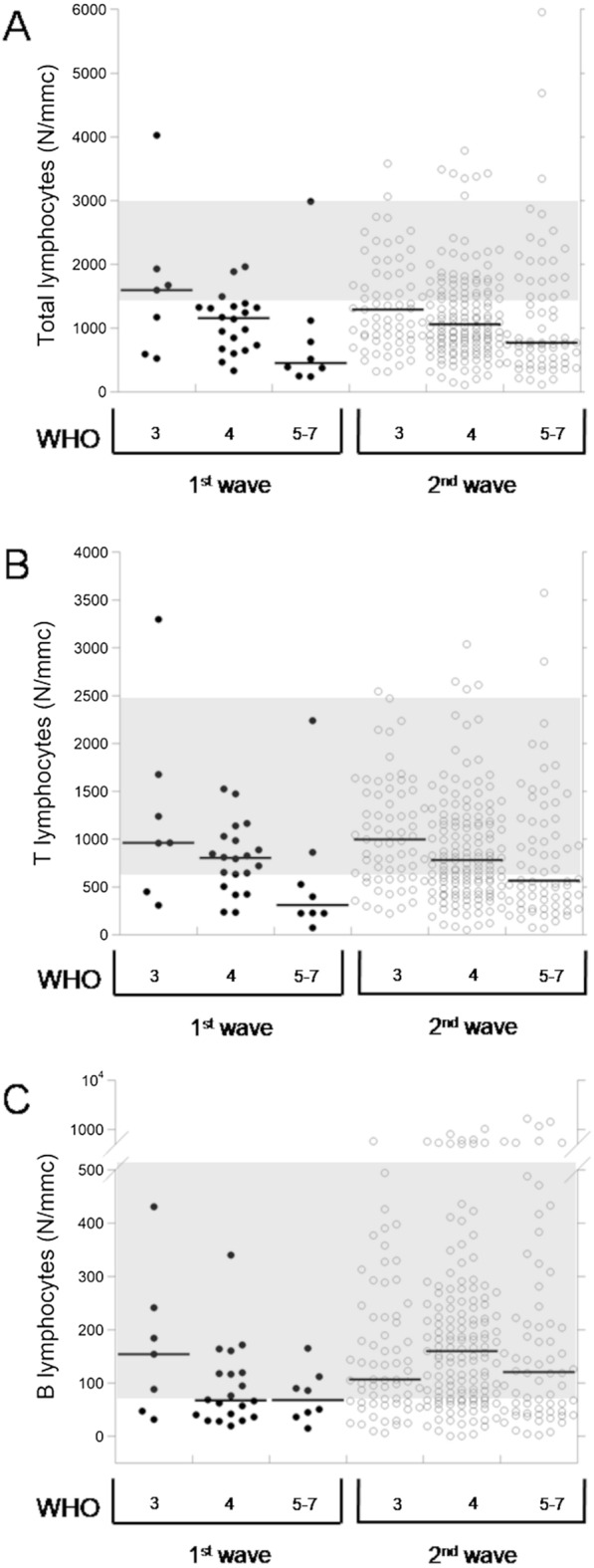
Dot-plots of total (**A**), T (**B**) and B (**C**) lymphocytes in 35 patients of COVID-19 1st wave and 274 patients of the 2nd wave at hospital admission. Gray areas indicate the reference ranges. Black lines indicate median values

**Fig. 2:**
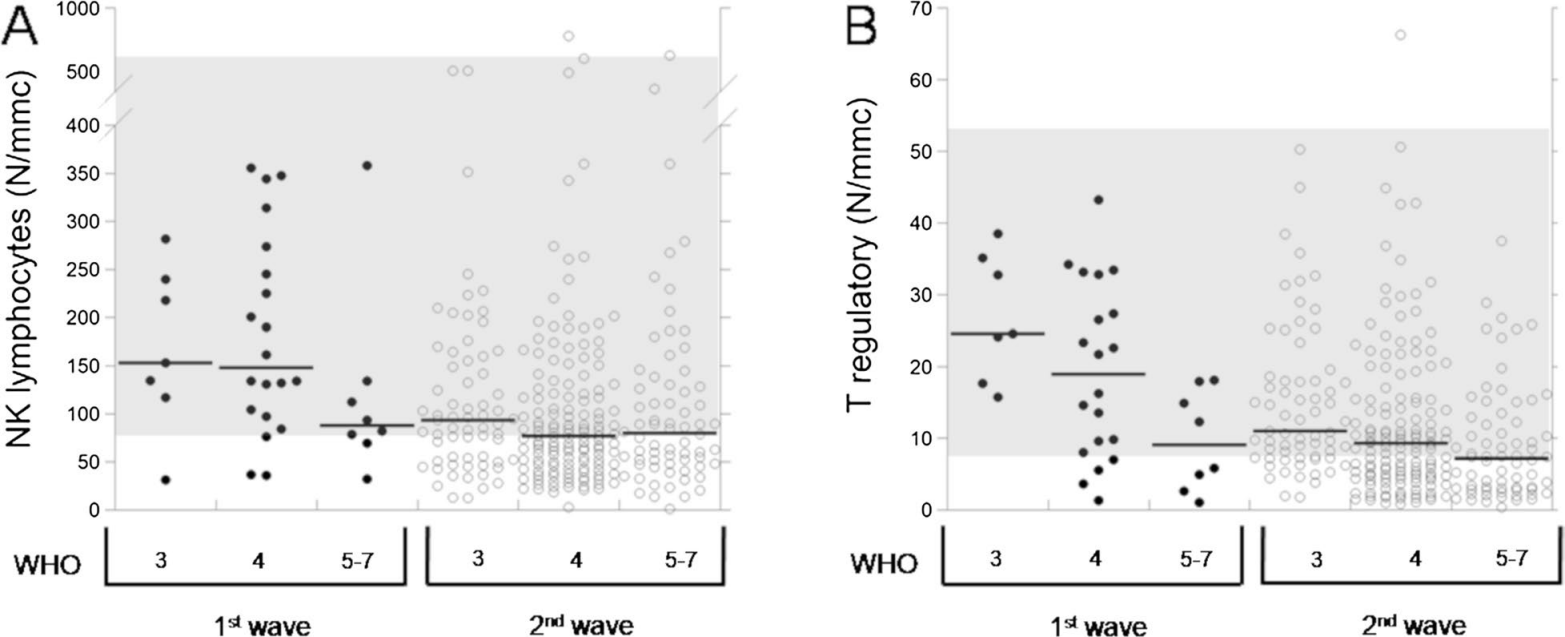
Dot-plots of NK (**A**) and T regulatory (**B**) lymphocytes in 35 patients of COVID-19 1st wave and 274 patients of the 2nd wave at hospital admission. Gray areas indicate the reference ranges. Black lines indicate median values

**Fig. 3:**
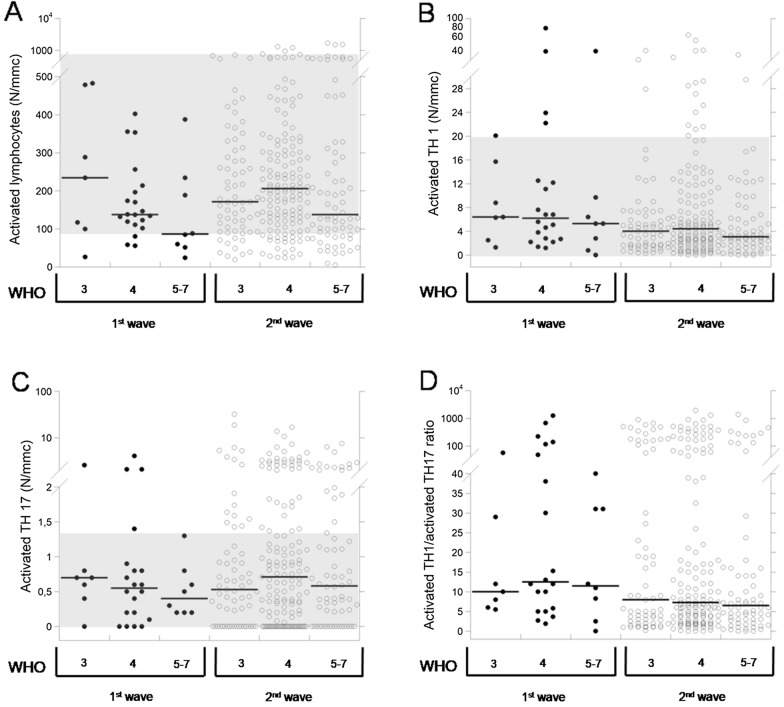
Dot-plots of total activated (**A**), activated TH1 (**B**), activated TH17 (**C**) lymphocytes and activated TH1/activated TH17 ratio in 35 patients of COVID-19 1st wave and 274 patients of the 2nd wave at hospital admission. Gray areas indicate the reference ranges. Black lines indicate median values

In addition, the data of serum IL-6 and lymphocyte subpopulations in 274 COVID patients of the 2nd wave in comparison with the 35 COVID patients of the 1st wave [3] are reported in Table 2. We describe the comparison of the data among the WHO subgroups of patients of the same wave as well as the comparison between the two waves patients bearing to the same WHO subgroup. Comparing the patients of the two waves, in patients of the 2nd wave we observed that:

- (i) Serum IL-6 was significantly lower in each WHO subgroup, with no differences among WHO stages. While, in patients of the 1st wave the levels of the marker showed an increasing trend with the stage [3].
- (ii) Total lymphocytes (Fig. 1A) were lower in patients of the WHO stage 3 and 4, while they resulted higher (always not significantly) in patients of the WHO stages 5–7. Furthermore, the trend of reduction with the stage was less pronounced than in patients of the 1st wave.
- (iii) T lymphocytes (Fig. 1B) were not different in patients of the WHO stages 3 and 4, while they resulted higher (although not significantly) in patients of stages 5–7. Furthermore, the trend of reduction with the stage was less pronounced than in patients of the 1st wave. T helper and T suppressor lymphocytes were lower in patients of the WHO stage 3, while they resulted not different in patients of the WHO stage 4 and higher (although not significantly) in patients of the WHO stage 5–7. Furthermore, for both the parameters we observed the same significant reduction with the progression of the WHO stage observed in patients of 1st wave [3].
- (iv) B lymphocytes (Fig. 1C) were lower in patients of the WHO 3 stage, while they resulted higher in patients of the WHO stage 4 (significantly) and of the stage 5–7. Furthermore, B lymphocytes did not show the trend of reduction with the severity that we observed in patients of the 1st wave [3]. Naïve lymphocytes were slightly lower in patients of the WHO stage 3 and higher in patients of the WHO stages 4 and 5–7 (although not significantly). Furthermore, the trend of reduction with the stage was less pronounced than in patients of the 1st wave.
- (v) NK lymphocytes (Fig. 2A) resulted lower in patients from all WHO stages (significantly for the WHO 4 subgroup) with no differences between subgroups, while in patients of the 1st wave they gradually reduced with the progression of the WHO stage (Fig. 2A). T regulatory lymphocytes (Fig. 2B) were lower in each WHO stage (significantly in subgroups 3 and 4), with the same trend of significant reduction with the increase of the WHO stage.
- (vi) Total activated (Fig. 3A), as well as activated T lymphocytes, were lower in patients of the WHO stage 3, significantly higher (total activated) or equal (activated T) in WHO stage 4, and both increased (although not significantly) in patients of the WHO stage 5–7.
- (vii) Both TH1 and TH17 lymphocytes were lower in WHO stage 3, equal in WHO stage 4 and higher (although not significantly) in WHO stage 5–7. Both the subpopulations had a trend of reduction with the stage (significant for TH1) in both 1st wave and 2nd wave.
- (viii) Activated TH1 (Fig. 3B) lymphocytes were lower (although not significantly) in patients of all WHO stages, while activated TH17 lymphocytes (Fig. 3C) resulted higher (although not significantly) in all WHO stages and did not show any trend with the increase of the WHO stage. Furthermore, we evaluated the ratio between activated TH1 and TH17 lymphocytes in patients of both the waves (Fig. 3D).

Finally, we performed the linear regression analysis to evaluate the effect of age, gender and pre-recovery treatments on the values of lymphocyte populations (Table 3). Both the age and the male gender were negatively related to the number of most lymphocyte subpopulations (i.e., total, T, T helper, T suppressor, naïve, T regulatory and TH1 lymphocytes). Furthermore, we observed a negative correlation between the assumption of steroids and the number of T suppressor, NK and T regulatory lymphocytes, and a negative correlation between the assumption of azithromycin and T, T regulatory and TH1 lymphocytes number.

**Table 3:** Linear regression analysis in 2nd wave COVID-19 patients

	Age		Gender (male)		Steroids*		Azithromycin*	
	Slope	P value	Slope	P value	Slope	P value	Slope	P value
IL-6	− 0.180	0.064	− 0.078	0.255	− 0.054	0.324	− 0.055	0.322
Total	− 0.206	** < 0.0001**	− 0.133	**0.015**	− 0.097	0.058	− 0.095	0.061
T	− 0.249	** < 0.0001**	− 0.170	**0.003**	− 0.096	0.059	− 0.113	**0.033**
T helper	− 0.212	** < 0.0001**	− 0.155	**0.006**	− 0.042	0.250	− 0.097	0.058
T suppressor	− 0.211	** < 0.0001**	− 0.115	**0.030**	− 0.113	**0.033**	− 0.068	0.134
B	− 0.089	0.075	− 0.045	0.232	− 0.013	0.414	− 0.005	0.468
Naïve	− 0.225	** < 0.0001**	− 0.121	**0.024**	− 0.100	0.052	− 0.084	0.086
NK	− 0.034	0.290	0.032	0.301	− 0.111	**0.035**	− 0.081	0.095
T regulatory	− 0.294	** < 0.0001**	− 0.211	** < 0.0001**	− 0.192	**0.001**	− 0.153	**0.006**
Total activated	− 0.094	0.064	− 0.058	0.175	− 0.028	0.323	− 0.012	0.422
Activated T	0.000	0.498	− 0.049	0.212	0.039	0.264	0.027	0.328
TH1	− 0.235	** < 0.0001**	− 0.224	** < 0.0001**	− 0.083	0.089	− 0.119	**0.027**
Activated TH1	0.040	0.259	− 0.027	0.332	0.043	0.245	− 0.016	0.395
TH17	− 0.078	0.102	0.052	0.201	− 0.006	0.463	− 0.085	0.084
Activated TH17	0.050	0.211	0.040	0.261	0.031	0.307	− 0.004	0.477
Activated TH1/TH 17	− 0.067	0.138	− 0.083	0.090	− 0.024	0.346	0.017	0.390

^*^Among the 274 COVID-19 patients of the 2nd wave, 161 have been treated with steroids and/or azithromycin before hospitalization

Significant values are reported in bold

## Discussion

Patients of the 2nd wave were significantly younger as compared with patients of the 1st wave, which have been described in our preliminary study [3], even if, in both the waves, the age of patients significantly increased with parallel to the WHO stage. Furthermore, among patients of the 2nd wave we observed a significantly higher percentage of females, although in advanced stages from both the waves the percentage of males was higher, indicating that age and male gender are risk factors for a more severe disease. In fact, the 10/12 (83.0%) patients of the 2nd wave, that died during hospitalization, were males (data not shown), in disagreement with a large Italian study that reported a higher percentage of females among patients died for COVID-19 during the 2nd wave [11].

Furthermore, in COVID-19 patients of the 2nd wave we observed a higher number of total, T, helper, suppressor, naïve and B lymphocytes in patients in advanced WHO stages (5 to 7), while in the other patients all (stage 3) or some (stage 4) of the above-mentioned subpopulations were lower in comparison to patients of the 1st wave of the corresponding WHO stage. These findings indicate that the 2nd wave was less severe only for patients in advanced stage, in disagreement with previous studies that reported a global less severe clinical impact of the 2nd wave of COVID-19. The younger age and the higher number of females that we observed in patients of the 2nd wave in comparison with patients of 1st wave, may depend on the improvement of organizational and diagnostic strategies that allow the early hospitalization of COVID-19 patients. The improvement might also depend on the steroid therapy performed before hospitalization (about 76% of patients of stages 5–7), while all patients of the 1st wave were hospitalized before starting therapies. Steroids could contribute to reduce the cytokine storm inhibiting the production of IL-6 [19, 20] thus rendering less pronounced the lymphocyte exhaustion [9], even if our regression analysis excluded the impact of steroid therapy on IL-6 serum levels.

While, the less encouraging results that we obtained in patients of WHO stages 3 (about 35% of which were treated before hospitalization) and 4 (about 70% treated before hospitalization) may depend on the prevalent inhibitory effect on lymphocyte proliferation that steroids and azithromycin exerted in patients with a less severe COVID-19 disease. In fact, the current literature reports that steroid therapy should be avoided in COVID-19 patients not progressed to a stage that requires oxygen support (like all our patients of WHO stages 3 and 4 before hospitalization), because the immunosuppressive effects of these drugs might hamper antiviral response [13, 19]. While, the pharmacological role of azithromycin is still questioned [21], given the weak evidences of its antiviral effect in COVID-19 patients [14]. In addition, all patients of the 2nd wave had a reduction of NK cells that have a relevant role in clearing virus-infected cells [8, 22] and of T regulatory lymphocytes. This reduction may depend on a higher number of T regulatory lymphocytes infected by the virus and thus removed [5], or on a higher number of cells migrated in lung [6], but may also depend on steroids assumed before hospitalization that inhibit the NK response [22] and reduce the T regulatory production [23]. The relevant reduction of T regulatory lymphocytes in COVID-19 patients was observed also in other studies [5]. Considering the immunomodulatory role of these cells in keeping in check inflammation, various therapeutic approaches have been proposed to stimulate the production of such cells [5]. Cytometric analysis may help to specifically address these therapies to patients with a lower number of T regulatory lymphocytes.

Furthermore, we observed a lower number of activated TH1 lymphocyte and an increase of activated TH17 cells particularly in patients with advanced WHO stages, despite the reduction of neutrophils [24] that should mediate TH17 promotion [25]. In addition, these results may be influenced by the therapies that our patients performed before hospitalization. In fact, it is known that both steroids [26] and azithromycin modify the TH axis suppressing activated TH1 and TH1 lymphocytes [27] and related chemokines like IFN-gamma impairing the antiviral response [28–30]. Thus, the TH1/TH17 balance is addressed toward a pro-inflammatory condition. Various studies suggested the inhibitory targeting of TH17, IL-17 or IL-17RA and the use of TH1 activators as potential therapeutic approaches for COVID-19 [31–35]. IL-17 inhibition has already been adopted as a common and successful strategy to reduce the injury associated with inflammatory autoimmune diseases including psoriasis and psoriatic arthritis. Dysregulation of TH17 cells and production of IL-17 in the skin, synovial space and endothelium promote the production of downstream pro-inflammatory molecules such as IL-1β, TNF and IL-6. Recruited neutrophils then produce IL-6 and reactive oxygen species, leading to characteristic skin lesions and joint destruction. In ARDS and in the acute lung injury, such us in psoriasis, there is a disruption of the balance of pro-inflammatory and anti-inflammatory cytokines [34]. Three commercially available anti IL-17 drugs exist: secukinumab (human monoclonal antibody to IL-17) [36], ixekizumab (humanized monoclonal antibody to IL-17) and brodalumab (human monoclonal antibody to the IL-17 receptor). Both secukinumab and ixekizumab are approved for psoriasis, psoriatic arthritis and ankylosing spondylitis; brodalumab is approved for the treatment of psoriasis alone [37]. By targeting IL-17, which operates ‘upstream’ of both IL-1 and IL-6 and results in a reduction of neutrophil recruitment, several factors known to play major roles in ARDS would be inhibited [34].

Our study demonstrates a strong variability of TH1/TH17 response among COVID-19 patients also within the same WHO stage. Thus, cytometric analysis may help to treat each patient with the most appropriate targeted approach by a monoclonal antibody treatment against the altered immune pathway, such as tocilizumab for IL-6 pathway [38] or secukinumab for IL-17 pathway [3, 33] alterations.

To conclude: our study indicates that in COVID-19 patients of the 2nd wave, the younger age, the prevalence of the females and steroid therapy before hospitalization have a global positive effect in patients with advanced WHO stages. While, the assumption of steroids and azithromycin at diagnosis has a negative impact on cytometric parameters in less severe patients worsening the immune response against the virus. A limitation of this study is represented by the low number of patients and the comparison between obviously non-homogeneous groups. Further studies are necessary to better define the therapies to be used in COVID-19 patients with different severity and to reveal prognostic biomarker that early predict the severity of the disease. In this context, cytometric analysis may contribute to select targeted therapies by monoclonal antibodies for each patient.

## Data Availability

All data used during the study are available from the corresponding author by request.
